# Remarks Made at the Anniversary Dinner of the American Academy of Dental Science in Boston, November 11, 1896

**Published:** 1897-08

**Authors:** Jacob L. Williams


### REMARKS MADE AT THE ANNIVERSARY DINNER OF THE AMERICAN ACADEMY OF DENTAL SCIENCE IN BOSTON, NOVEMBER 11, 1896.

BY JACOB L. WILLIAMS, M.D.

Mr. President and Gentlemen,—I am very glad to hear the pleasant allusion to Dr. Tucker, who was one of the original members,—in fact, both Drs. Tucker, Joshua and Elisha, were original members of this Academy.

In 1867 some half-dozen or more of us—I was then rather a junior member of the profession—met, and, after talking over dental matters, thought we would have a little society of our own, believing that there would be mutual advantages in combination and union on the principles of charity, tolerance, sympathy, and mutual confidence. We formed that society—about half a dozen of us—twenty-nine years ago; to-day there are more than one hundred members on its rolls in excess of the original number.

But that is not all; I am glad to see, and I congratulate the Academy on the fact, that the objects as implied by the name and the general tone of that combination have been perpetuated and have grown with its increasing membership,—the desire for knowledge and perfection in one's own work and the willingness to share one's ideas instead of keeping them secret, and, further than that, in the matter of ethics, willingness to give credit to an idea and to the individual who originates an idea, which in those days was not so conscientiously considered as perhaps it is to-day. I remember an instance of this which has been touched upon this evening, and as it is a matter of history, there can be no harm in my referring to the subject again.

When Dr. Wells offered his anæsthetic to the Harvard Medical School he put up a notice in the Medical College stating that a *public* demonstration of the effects of this agent would be given on a certain day. Dr. Wells came, and the operation of tooth-evulsion was made on a patient while under the effects of nitrous oxide gas.

Dr. D. M. Parker, formerly a president of this society, was present and performed the operation, and has often asserted that the operation was as successful as the average under ether, although the subject, as is usual, made some demonstration, so that the students laughed at the matter and poor, modest Wells felt rather discouraged.

I can tell you in a few words the story of the origin and introduction of *ether* anæsthesia, about which there has been so much discussion; and I was in a position at the time to be thoroughly conversant with the matter. When it was first introduced I was a pupil of Dr. Keep. Dr. Wells had Morton as a pupil, and afterwards as a partner. Morton was inclined to extract teeth recklessly (as Dr. Cheever says he has formerly seen done), the main thought being to get out as many teeth as possible, as at that time he was doing quite a "business" in a kind of artificial work that was not held in high repute by the best members of the profession.

In a certain case where he wished to do something of this sort, he remembered that Dr. Wells had some knowledge of anæsthetics, and it occurred to him that if the patient by taking something could be put into a condition so that the teeth could be extracted without objection, he could have another case of that kind of business to do. So he went to Dr. Charles T. Jackson and asked if he could not prescribe something that would prevent pain in extraction of a tooth. Dr. Jackson was a man whose head was full of ideas that he was willing to give on application, and he recommended to Morton the use of sulphuric ether, which he had tried himself, at the same time giving him special directions for its use and cautions against carelessness. Morton took the idea and followed it as prescribed, with the predicted success; and having a plausible and persuasive ability, which is sometimes called the "bunco" talent, he persuaded Dr. Jackson to join with him and file a caveat for a patent, which was done, thereby acknowledging Dr. Jackson's originality of the idea. After thinking the matter over more calmly, Dr. Jackson decided that it was an unethical thing to do, and he withdrew from his arrangement with Morton, saying that he would have nothing to do with the patent. Morton determined to go on with the matter alone, and to take for himself whatever profit and honor he could get from it, so he patented this prescription of Dr. Jackson's, and then went to the head of the staff of the Massachusetts General Hospital, the venerable Dr. Warren, and said that he had discovered a compound that would destroy pain in surgery.

It is to the credit of Dr. Warren that it can be said that, no matter what the source of an idea which was claimed to have merit, he stood ready to investigate anything which was declared to be of value in the practice of medicine or surgery. The staff did investigate, and proved Dr. Jackson's prescription for themselves; after dashing aside the veil of chicanery and the patent shackles in which it was presented to them, they had the credit of endorsing it to the world, and I think very justly the present hospital staff should have celebrated the fiftieth anniversary of their introduction to the world of that anæsthetic, although it did not take up two years before the suggestion of Dr. Wells,—viz., the production of temporary anæsthesia by the use of nitrous oxide gas.

At the Pan-American Medical Congress, held in Washington in September, 1893, I sketched in a paper some facts in regard to the origin of the application of anæsthesia in surgery, referring to Dr. Long, of Georgia, who had used ether in his private practice some years before, and Colton, who went about the country showing the amusing effects of nitrous oxide gas, from which Dr. Wells took the idea of using it for surgical anæsthesia, also to Sir Humphry Davy's long previous suggestion as to the possibility of doing something of this kind.

Now, the point is, whether the physician who originates an idea, or an ignorant nurse who carries it out, or the person who appropriates it, patents it, and calls it his own, is entitled to the credit that may accrue from the use of that idea? In ethics and by common law a physician has the credit and the responsibility for his prescription.

As I have stated, to the hospital staff belongs the credit of testing and endorsing to the world the value of Dr. Jackson's prescription, notwithstanding the false disguise in which it came to them. These are the main facts of the case, and I was sorry not to see the physician, Dr. Jackson's, name who gave the original prescription introduced in the celebration.

This Academy has been fortunate in being composed, in the main, of members who have strictly conformed to the principles of ethics. I do not know of an instance where any of its members have obtained an idea at our meetings and advertised it to the world as theirs, and I trust we shall always be as fortunate in our members.

Then, again, the matter of saving teeth, which has been alluded to, we have also grown in that respect, and our studies and investigations have led us to consider the teeth as being valuable not only in themselves, but to the general economy.

At a meeting of the Oral Section of the American Medical Association more than a year ago, during a discussion of this subject, a suggestion was made by some speaker that physicians and surgeons were regardless of the value of teeth. I could not help getting up and saying that I had known that to be a fact in past years, but my acquaintance and experience had led me to believe that at present well-informed surgeons were fully cognizant of the value of the teeth and the importance of saving them, and that they paid vastly more regard to them than formerly (and this has been evidenced here to-night by Dr. Cheever's remarks); but, said I, we have to "look at home;" there are to-day many so-called dentists who have not yet conscientiously learned this important fact, and who still rake out and cut off useful teeth for the sake of putting in their artificial appliances. That work is still going on. I am glad to say that I do not think there is a member of this society who would not pronounce it malpractice to destroy a useful organ for the sake of having an opportunity of replacing it with artificial work. I often think in that respect of one of Dr. Holmes's poems which has always delighted me, "The Two Armies," and the lines which seem to express my own feeling in this matter run in this way:

"One marches to the drum-beat's roll,
The wide-mouthed clarion's bray;
And bears upon a crimson scroll,
'Our glory is to slay!'

"One moves in silence by the stream,
With sad yet watchful eyes;
Calm as the patient planet's gleam
That walks the clouded skies;

"Along its front no sabres shine,
No blood-red pennons wave;
Its banner bears the single line,
'Our duty is to save!'"

That, I think, represents the tone of this Academy, and I am glad to see it and hear it, and while one might expect it from the *personnel*, I still wish to congratulate the society on its present condition of advancement.

